# CEO Regulatory Focus, Analysts’ Optimism Bias, and Firm Strategic Change: Evidence From Chinese-Listed Companies

**DOI:** 10.3389/fpsyg.2022.813920

**Published:** 2022-03-24

**Authors:** Chun Huang, Wangxiongjie Zheng

**Affiliations:** College of Management, Zhejiang University of Finance and Economics, Hangzhou, China

**Keywords:** CEO regulatory focus, firm strategic change, analysts’ optimism, CEO prevention focus, optimism bias

## Abstract

With the ongoing coronavirus disease 2019 (COVID-19) pandemic, technological, socio-political, and institutional changes have led to a “new normal” competitive landscape, firms must make longer-term strategic changes to deal with short-term discontinuities and great uncertainties to acquire sustainable advantage. Based on regulatory focus theory and upper echelons theory, this study explores the relationship between CEO regulatory focus and corporate strategic change and examines the moderating effects of analysts’ optimism bias in earning forecasts. The study uses data from A-share-listed companies in China during 2010–2018. We find that CEO promotion focus is positively associated with strategic change, while CEO prevention focus is negatively associated with strategic change. We also find analysts’ optimism bias in earning forecasts would moderate these relationships.

## Introduction

The coronavirus disease 2019 (COVID-19) pandemic will further exacerbate existing technological, socio-political, and institutional changes, and lead to a “new normal” competitive landscape; firms must make longer-term strategic changes to deal with short-term discontinuities and great uncertainties to acquire sustainable advantage (Hitt et al., 2021). In this case, managers need to respond quickly and formulate new and more flexible strategies to improve corporate strategic adaptability (North, 1999; Hitt et al., 2021). Therefore, it is necessary to fully explore the consequences of the strategic change to the enterprise and identify the boundary conditions at this stage.

Strategic change represents a central concern in strategic management research, and previous studies have attempted to understand the antecedents that may influence the corporate strategic change—an organization adjusts its key resource allocation to adapt to the external environment (Adner and Helfat, 2003; Gedajlovic et al., 2004; Boyne and Meier, 2009). As part of these works, recent research has drawn from the upper echelon theory. For example, Zhang and Rajagopalan (2010) proposed that the origin of CEOs will significantly affect the relationship between strategic change and corporate performance; Nadkarni and Herrmann (2010) based on the Big Five personality model explored the impact of CEO personality characteristics on strategic change.

However, in the research stream of the impact of CEO characteristics on strategic change, an important psychological motivation that shapes the CEO decision-making is noticeably absent; it is CEO regulatory focus. The regulatory focus theory advocates that individuals are stimulated by two independent regulatory focuses, which are the promotion focus and the prevention focus (Higgins, 1997). Individuals with a high promotion focus pay attention to the “ideal self,” pursue the maximization of benefits as the goal, and prefer to take risks (Lanaj et al., 2012). On the contrary, individuals with a high prevention focus pay more attention to “ought self” and are willing to make and follow rules to avoid making mistakes as their main goal (Lanaj et al., 2012). Both promotion focus and prevention focus can independently shape individual goal-seeking tendencies and their specific strategic means (Crowe and Higgins, 1997; Lanaj et al., 2012). Thus, CEO regulatory focus could have a considerable effect on corporate strategic changes (Gamache et al., 2020). Building on this, to make a useful supplement to existing strategic leadership research, this article explores the relationship between CEO regulatory focus and corporate strategic changes.

At the same time, numerous studies have proved the important role of external governance bodies in strategic decision-making (Gedajlovic et al., 2004; Zhang and Rajagopalan, 2010). There are two main types of corporate external governance entities. One can directly affect corporate strategic decisions (institutional investors and regulators, etc.), and the other can cause indirect impact on corporate strategic decisions (analysts and the media, etc.). However, scholars have not considered how external governance entities indirectly influence the relationship between CEO individual characteristics and the firm’s strategic behavior. As an important stakeholder that may cause indirect impact on the relationship between CEO characteristics and corporate strategic change, analysts’ forecast can be a considerable mean for external investors to understand the company, which can effectively reduce the information asymmetry between the company and the market, and eventually affect CEOs’ ability to formulate and implement strategic changes through stock price fluctuations and financing constraints. Therefore, to further explore the above research gap, we chose analysts’ optimism bias as our moderating variable in this study. Accordingly, we examined how the relationships between the two CEO regulatory focus and the level of strategic change differs with the level of analysts’ optimism bias in earning forecasts.

Our work makes three contributions. First, this study further expands the research framework of upper echelons theory by introducing the regulatory focus theory into the research context of the influence of CEO individual psychological characteristics on corporate strategic decisions. Previous pieces of research have noted that understanding the psychological mechanisms that drive executive behavior is important for future upper echelons theory research (Hambrick, 2007; Chiaburu et al., 2010). However, CEO regulatory focus is rarely studied in the field of upper echelons theory research. Specifically, we find that CEO with stronger promotion focus would be more inclined to make corporate strategic change, and CEO with stronger prevention focus would be less inclined to make corporate strategic change, which extends strategic consequences study of CEO regulatory focus.

Second, the development of our empirical model makes contribution to the research of antecedents of strategic change. Previous scholars did numerous works to examine the antecedents of organizational strategic change from the perspective of cognition (Boeker, 1997; Barker et al., 2001). However, as an important influencing factor of individual behavioral motivation, regulatory focus has not been discussed and studied as a cognitive antecedent of strategic change. Based on the regulatory focus theory, our research explores the impact of CEO regulatory focus on corporate strategic change, which informs the recent stream of research that strives to understand the antecedent of strategic management.

Finally, we contribute to the literature of contextualized strategic leadership research by introducing the analysts’ optimism bias to our empirical model. External governance entities can exert a “pull” or “push” force in the influence of executives’ personalities, values, and motivations on strategic decisions. However, an analyst as an important external stakeholder that will affect corporate strategy indirectly has not been seriously considered on the strategic leadership research. This study chooses analysts’ optimism bias in the earning forecasts as our moderating variable to explore its contextual effect on the relationship between CEO regulatory focus and corporate strategic change. Our findings speak to the theorized importance of analysts’ behavior in the process of CEO decision-making.

## Theoretical Background and Hypothesis Development

### CEO Regulatory Focus and Strategic Change

#### Strategic Change

Corporate strategic change is the adjustment of a firm’s resource allocation pattern in response to environmental change (Mintzberg, 1989; Zhang and Rajagopalan, 2010). Research on the corporate strategic change could be mainly divided into two groups: the first one is the performance implication of corporate strategic change, and the second is the antecedents of corporate strategic change. To find the antecedent of strategic change, previous studies have used the multiple lens to examine organizational (e.g., resources and competencies) and environmental (e.g., local competition, regulatory changes, technological changes) antecedents of strategic change initiation (Zajac and Kraatz, 1993; Johnson and Steinman, 2009).

In fact, in this research stream, a large number of studies are based on upper echelon theory, exploring the impact of the top management team and CEO personal characteristics on corporate strategic changes (Gioia and Chittipeddi, 1991). For example, from the level of the top management team, some scholars explore how the top management team’s age, tenure, and educational background affect how they perceive and interpret the external environment, and ultimately affect the process mechanism of strategic change (Rajagopalan and Spreitzer, 1997; Brockner et al., 2004). Another group of scholars studies the impact of CEO personal characteristics (e.g., origin, tenure, personality, and succession) on corporate strategic changes (Tumasjan and Braun, 2012). Although the characteristics of the CEO and the top management team in the above studies all show a certain degree of relevance to corporate strategic changes, these characteristics are mostly static and do not reflect the process mechanism of how CEO individual characteristics affect corporate strategic change. Therefore, it is extremely necessary to start from the perspective of motivational theory. Regulatory focus theory posits decisions and actions of individuals through two motivational systems to regulate which are promotion focus and prevention focus. Hence, exploring the relationship between CEO regulatory focus and corporate strategic change could make us have a deeper understanding of the dynamic mechanism of the CEOs’ influence on corporate strategic change.

#### Regulatory Focus Theory

The regulatory focus theory, first proposed by Higgins in 1997 [8], aims to reveal the mechanisms underlying people’s approach-avoidance motivation. The theory suggests that individuals have both nurture-related regulation and safety-related regulation in the process of survival, with growth-related regulation containing promotion focus and safety-related regulation containing prevention focus. People differ in their choice to approach pleasure as well as to avoid pain and injury. To discover the true nature of the approach-avoidance motivation, psychologists need to look beyond the hedonic principle to a different perspective on the mechanisms at work. One important principle is the regulatory focus, which includes two focuses; they are promotion focus (achievement and passion) and a prevention focus (safety and responsibility).

Specifically, the promotion focus helps individuals meet growth needs by guiding them to adopt convergent strategies to pursue desired goals (e.g., ideals, aspirations, ambitions, etc.), achieve desired states through the pursuit of success, and thereby acquire progress, growth, and achievement. And the prevention focus helps individuals to meet their security needs and guides them to adopt avoidance strategies, pursue responsibility goals (e.g., duty, responsibility, etc.), and achieve the desired state by avoiding failure. The two different regulatory foci guide individuals to differences in need orientations and strategies (Johnson et al., 2017). And the two regulatory focuses are coexisting and independent, driven by different neurocognitive systems and have low correlations with each other; people may have only one high degree of regulatory focus, two high degrees of regulatory focus, or even two lower degrees of regulatory focus at the same time (Lanaj et al., 2012). Therefore, for the CEOs, higher in promotion focus or prevention focus can have certain implication for a firm’s strategy (Gamache et al., 2015; Johnson et al., 2015; Kammerlander et al., 2015). We extend this research by exploring the relationship of CEO regulatory focus and the level of corporate strategic change.

#### CEO Promotion Focus and Strategic Change

Applying regulatory focus theory to the strategic change study, we argue that CEO with strong promotion focus would actively engage in corporate strategic change. Firstly, promotion-focused CEOs are more motivated by strategic change in an organization. Promotion focus makes individuals more focused on achievement, passion, and motivation out of the need for growth and progress (Crowe and Higgins, 1997; Brockner et al., 2004) and a strong sense of purpose for what they are doing (Lanaj et al., 2012). At the same time, promotion-focused CEOs show an extreme concern for achievement and reward (Lanaj et al., 2012; Gamache et al., 2015) and a high demand for scale and volume of output (Brockner et al., 2004). Strategic change, however, is an important means by which companies can appropriately respond to the changing and evolving external environment and is closely related to the achievement of their development vision and performance goals. Therefore, it can be argued that promotion-focused CEOs will make strategic changes for the purpose of goals such as increasing the size of the firm or expanding the firm’s market share.

Additionally, promotion regulatory focus motivates individuals to more actively explore potential opportunities and make positive assessments of potential risks (Gamache et al., 2015). The high correlation with exploration orientation suggests that promotion-focused CEOs will seek out potential change opportunities in the firm whenever possible (Friedman and Förster, 2005). Promotion focus motivates individuals to assess the environment from the perspective of seeking opportunities (Higgins, 1997). For example, Tumasjan and Braun (2012) found that promotion focus was positively related to entrepreneurs’ ability to identify opportunities. Promotion-focused CEOs similarly assess opportunities positively (Gamache et al., 2020). Moreover, promotion focus makes individuals perceive potential gains more than potential losses (Crowe and Higgins, 1997; Brockner et al., 2004), driving individuals to be sensitive to the positives of the status quo (Lanaj et al., 2012). When CEOs consider whether to make a strategic change decision, the promotion focus causes them to focus more on the positive consequences of strategic change. Thus, promotion-focused CEOs will focus more on the positive effects of strategic change and environmental adaptation and perceive the disruptive nature of strategic change as positive. Such CEOs will facilitate strategic change by focusing on the potential collaboration possibilities associated with future change success, optimistic forecasts, and market assessment (Wowak and Hambrick, 2010).

Finally, promotion focus is strongly related to the “ideal self” (Higgins, 1997; Friedman and Förster, 2005), and promotion-focused individuals tend to “prevent errors of omission” (Crowe and Higgins, 1997). Even though the outcome of a strategic change is highly uncertain, the promotion-focused CEO will be guided by the principle of “leaving no stone unturned” and will be ambitious about the decisions he or she makes (Gamache et al., 2020). The optimistic nature will eventually be externalized to the behavior of the promotion-focused CEO, as Gamache found that promotion-focused CEOs seek goal achievement through a wide range of potential internal and external opportunities and have greater willingness to initiate corporate mergers and acquisitions and stakeholder strategies (Gamache et al., 2020). Therefore, as an important factor for firms to achieve their own strategic goals, promotion moderating focus CEOs will be more likely to make decisions for strategic change. In summary, according to the regulatory focus theory, promotion focus CEOs will tend to implement corporate strategic change in terms of decision motivation, cognitive patterns, and behavioral styles. Therefore, the following hypotheses are proposed.

**H1: CEO promotion focus is positively associated with the level of corporate strategic change.**

#### CEO Prevention Focus and Strategic Change

According to the regulatory focus theory, we propose that CEO prevention focus would be negatively associated with the level of strategic change. First, CEOs with a strong prevention focus place more emphasis on their duties and responsibilities to shareholders in corporate governance and emphasize employees’ trust in their own leadership (Higgins, 1997; Kammerlander et al., 2015; Kark and Van Dijk, 2019; Gamache et al., 2020). As a result, CEOs with a strong prevention focus will pay more attention to their job and prioritize gaining the trust of the company’s shareholders, employees, and other stakeholders (Gamache et al., 2015). In fact, prevention regulatory focus CEOs will seek to find an explanation for their behavior as much as possible out of a sense of responsibility to shareholders (Kammerlander et al., 2015; Cuculiza et al., 2020), while strategic change will create a high degree of uncertainty (Zhang and Rajagopalan, 2010). Therefore, from a motivational point of view, CEO with higher prevention regulatory foci will not be inclined to make strategic changes for the sake of corporate governance interpretability (Gamache et al., 2015).

Second, prevention focus individuals are highly alert to making mistakes (Lanaj et al., 2012). CEOs with a strong prevention focus are driven by security and responsibility, make decisions with great care, and are sensitive to negative information (Brockner et al., 2004; Wallace et al., 2010). This can be reflected in their cautious and systematic approach to decision-making and intense due diligence. A prevention focus can also deter people from going astray (Crowe and Higgins, 1997). Thus, when making a strategic change, prevention-focused CEOs will be more concerned with the impact of a misguided strategic change than with missing a good change opportunity. Wowak and Hambrick argue that prevention-focused CEOs will be more concerned with resource integration issues and the lack of relevant domain experts (Wowak and Hambrick, 2010). These concerns suggest that prevention-focused CEOs will try to avoid strategic changes that carry high risks.

Third, prevention regulatory focus individuals seek rules and accuracy (Förster et al., 2003; Förster and Higgins, 2005). To make strategic decisions that are accountable to shareholders and prevent failures at the management level, corporate governance often requires well-developed rules and regulations and a rigorous and responsive board of directors (Daily and Dan, 2003; Sonnenfeld et al., 2013). They ensure that the board’s oversight status is maintained and actively communicate with the board to keep the board and shareholders informed and to seek advice on strategic actions to be taken by the firm (Sonnenfeld et al., 2013). In fact, both good monitoring and effective communication are effective ways to reduce agency costs (Förster et al., 2003). In contrast, strategic change often implies changes in multiple aspects of the firm, and it may reduce the firm’s resource allocation efficiency and require higher agency costs (Zhang and Rajagopalan, 2010). Therefore, from the perspective of the behavioral decision-making style of prevention focus CEOs, it can be argued that prevention focus CEOs are negatively related to corporate strategic change.

Based on the above analysis, this study proposes the following hypothesis.

**H2: CEO prevention focus is negatively associated with the level of corporate strategic change.**

### The Moderating Role of Optimism in Analysts’ Earning Forecasts

Numerous studies have proved the important role of external governance bodies in strategic decision-making (Gedajlovic et al., 2004; Zhang and Rajagopalan, 2010). There are two main types of corporate external governance entities. One can directly affect corporate strategic decisions (institutional investors and regulators, etc.), and the other can cause indirect impact on corporate strategic decisions (analysts and the media, etc.) (Hong et al., 2000). However, scholars have not yet considered how external governance entities indirectly impact relationship between executive characteristics and corporate strategic behavior. Therefore, we chose analysts’ optimism bias as our moderating variable to further explore the above research gap. Because analysts are often influenced by their own subjective emotions and objective external environment when assessing corporate earnings and writing related forecasts, and there may even be “complicity” between insiders and analysts, bias in analysts’ forecasts is widespread (Dowen, 1989; Ajinkya et al., 2005). For this reason, “analyst forecast bias” has received much attention from scholars (Eggers and Kaplan, 2009). Many studies have shown that analyst forecast bias affects firms’ exploratory behavior, innovation decisions, and input levels, and even firm risk taking (Cuculiza et al., 2020). This is because analyst forecasts are an important means for external shareholders of the firm to understand the firm, which can effectively reduce the information asymmetry between investors and the firm internally, and facilitate investors’ opinions on corporate governance and ultimately influence the strategic choices of the firm (Chen et al., 2010).

Therefore, given the different direct effects of the two CEO regulatory foci on strategic change, the following content examines the moderating mechanism of analysts’ forecast optimism bias in two paths: first, as mentioned earlier, strategic change is highly uncertain (Zhang and Rajagopalan, 2010), and external stakeholders will also present an uncertain attitude toward corporate strategic change (Chen and Jiang, 2006). Therefore, the CEO will bear the pressure of future expectations and financing constraints from external stakeholders. However, this constraint on the CEO’s decision will be reduced due to the existence of optimistic bias in analysts’ earning forecasts. It can reduce the information asymmetry between external investors and the firm in some extent, making external investors more optimistic about the firm’s future performance and more willing to invest (Bali et al., 2017; Cho et al., 2019). Furthermore, due to the herding effect of analysts, investors can be unanimously optimistic in the short term, which will cause more external funds to flow into the company, thereby reducing the financing constraints faced by the company when making strategic changes, which, in turn, reduces the pressure of financing constraints faced by the firm when making strategic changes (Alexander and Thomas, 2014). Therefore, when the CEO with higher promotion regulatory focus is trying to push the corporate strategic changes, the greater of the analysts’ optimism bias in earning forecasts, the more likely the CEO will carry out corporate strategic changes.

Second, the greater the optimism bias in analysts’ earning forecast, the more cautious the prevention focus CEO will be in making strategic changes. In fact, analysts often find it difficult to stick to their own views, and their forecasts tend to follow the changes in the market, i.e., there is a “change of face behavior.” Moreover, analysts often follow the herd blindly, showing obvious herd characteristics (Gao, 2008). There is a “beauty contest effect” in analysts’ forecasting behavior; analysts tend to consider the expectations of other participants in the market and their expectations of other participants when making forecasts, and do not objectively make optimal forecasts based on their own models (Sofus, 2008). Just as in a beauty pageant, the judges tend to make choices that are close to the average preference of the entire selection. Such analysts’ optimistic forecast bias often leads to greater stock price volatility and operational risk for companies. Previous studies have pointed out that analysts’ forecast optimism bias will increase the risk of stock price collapse (Cho et al., 2019). In contrast, CEOs with a strong prevention focus are sensitive to error and the pursuit of rules and stability (Higgins, 1997), and the volatility and vulnerability of stock prices and business risks associated with analysts’ optimistic forecasts often “deter” them. Therefore. CEOs with a strong prevention focus will be more cautious in making strategic changes in the face of optimistic deviations in analysts’ forecasts. This leads to the following hypothesis:

**H3: Analyst optimism bias in earning forecasts would amplify the positive relationship between CEO promotion focus and the level of strategic change.**

**H4: Analyst optimism bias in earning forecasts would amplify the negative relationship between CEO prevention focus and the level of strategic change.**

## Materials and Methods

### Sample and Dataset

2010 is the first year of the 2010s, and the global economy is slowly recovering from the 2008 economic crisis. Also, 2010 is the first year that China became the world’s second largest economy. After that, Chinese companies are facing a new era of major development, major adjustments, and major changes. In order to cope with the changes and challenges of the internal and external environment, strategic changes are crucial for Chinese companies at this time. Therefore, we chose 2010 as the starting point of the sample interval, testing our hypotheses on a sample of firms listed on the Shanghai Stock Exchange and Shenzhen stock exchanges from 2010 to 2018. We collected financial data from the CSMAR (China Stock Market and Accounting Research) database. To measure the CEO regulatory focus, we followed Gamache et al. (2015) and analyzed “Analysts and Discussion of Management Level” in Corporate Annual Report. Previous research on CEO regulatory focus has argued that changes in strategic decisions are a common consequence to the CEO regulatory focus. In view of this, this study defines, identifies, and measures strategic change by using CEO regulatory focus as an event.

The sample selection process of this study is specified as follows: Firstly, selecting firms whose CEOs were inaugurated in 2010-2018 and served for more than 3 years, 576 firms remained; secondly, excluding firms with missing CEO regulation focus. Due to the lack of management analysis and the discussion section in some annual reports and the fact that some companies have too little narrative in this section, the final number of companies that passed the screening process was one. Finally, excluding companies with missing data on the level of corporate strategic change, as some companies’ financial data (e.g., R&D investment data) were not disclosed, so the final valid sample of companies in this study was 387; the total sample size is 2,284 observations.

### Measures

#### Dependent Variable

Zhang et al. proposed that the average difference of the six strategic key indicators of advertising intensity, R&D intensity, plant and fixed equipment renewal ratio, non-production expense ratio, survival ratio, and debt ratio can be used to measure strategic change (Zhang and Rajagopalan, 2010). These 6 variables represent relatively independent resources that the CEO can control and deploy. Due to the poor availability of data on the update rate of plant and fixed equipment of listed companies in China, this indicator was changed to the fixed asset rate. The data of the above 6 indicators come from the CSMAR database. In addition, studies have shown that the CEO’s substantial impact on the company’s strategy began in the 2nd year (t) of his tenure. In addition, some studies have found that it usually takes 2 years for most companies to complete major changes (Abernethy et al., 2020). Therefore, this study chooses the 4th year of the CEO’s tenure (t + 2) to measure the results of corporate strategic changes. In order to exclude industry influence, the final data are obtained by subtracting the industry median from the average of the above indicators. In order to ensure the stability of the data, the industry median is calculated after excluding the top four companies in the industry. Finally, the difference between the average value of the six indicators adjusted by the industry median in the 4th year (t + 2) and the current year (t − 1) is recorded as the level of strategic change. For example, if the CEO of a company took office in 2010, the average value of the company’s 6 indicators in 2010 is recorded *s*_*t–1*_, and the median of the company’s industry in 2010 is recorded as *m*_*t–1*_. The average value of the six indicators after the number adjustment is recorded as *s*_*t*−1_−*m*_*t*−1_, the measurement year of the strategic change result is 2013, and the average value of the other six indicators is recorded as *s*_*t+2*_. The number of digits is denoted as *m*_*t+2*_, and the mean value of the 6 indicators after the median adjustment is denoted as *s*_*t* + 2_−*m*_*t* + 2_. Therefore, the strategic change is *S* = *s*_*t* + 2_−*s*_*t*−1_−*m*_*t* + 2_*m*_*t*−1_.

#### Independent Variable

Regulatory focus is often independent of self-subjective consciousness, and implicit or indirect measurement methods are the most effective (Johnson et al., 2012a,b; Lanaj et al., 2012; Mount and Baer, 2021). Previous studies have measured this variable mainly through text analysis of shareholder letters in the annual reports of listed companies (Gamache et al., 2015). As an important way for the CEO to communicate and report with shareholders in the annual report, the letter to shareholders has the characteristics of consistency and comparability. It is an ideal study for longitudinal research (Johnson et al., 2015). However, since “letters to shareholders” are relatively rare in A-share-listed companies, previous studies often used “Management Discussion and Analysis” in annual reports for replacement. Therefore, we reviewed the “Management Discussion and Analysis” that appeared in the annual reports of A-share-listed companies. Text analysis is carried out in the “Analysis and Discussion of Business Conditions” section. It is worth mentioning that, since the regulatory focus of subordinates is often shaped by the regulatory focus of the leader (Lanaj et al., 2012; Kammerlander et al., 2015), it can be considered that, even if other executives are involved in writing, the final point of view and the way of writing will still reflect the CEO’s own willing.

The following are the text analysis steps: First, use the scrapy package of Python3.7 to crawl the annual report of Juchao Information Databse, and get a total of 2,554 annual reports; second, extract the text data in the annual report through PDFFileReader, and clean the full text; third, refer to the word dictionary developed in previous studies (Gamache et al., 2020), which has 27 promotion words (accomplish, achieve, expand, etc.) and 25 prevention words (safety, risk, fear, etc.), and we performed our content analysis by using Jieba word segmentation. Sofus pointed out that it is simple, objective, reproducible, and transparent to analyze text information by counting “word count”; that is, word frequency (Finkelstein and Hambrick, 1990; Pitcher et al., 2000; Barker et al., 2001; Wu et al., 2007; Tuncdogan et al., 2016). Therefore, in the end, we measured the CEO promotion focus and the CEO prevention focus by measuring the word frequency of the promotion-related keywords and prevention-related keywords in the corresponding parts of the annual report.

#### Moderate Variable

Drawing on existing research (Eggers and Kaplan, 2009; Chen et al., 2010; Alexander and Thomas, 2014), the following formula is used for this article to construct FERROR, a measure of the optimistic deviation of analyst earnings forecasts.


$$FERROR=\frac{\sum FEPS-\sum EPS}{\sum EPS}$$

Among them, FERROR represents the degree of optimistic deviation of analysts’ earnings per share forecast for the year. FEPS represents the predicted value of earnings per share; EPS represents the actual value of earnings per share. When the FERROR value is positive, it indicates that there is an upward forecast bias, and analysts’ earnings forecasts are more optimistic. Similarly, when FERROR is negative, it indicates that there is a downward forecast bias; analysts’ earnings forecasts are more pessimistic. The larger the value of FERROR, the higher the degree of optimism of the analyst’s earnings forecast, that is, the more optimistic the analyst’s sentiment is.

#### Control Variable

To control the possible confounding variables that may affect the CEO regulatory focus and strategic change, we controlled the following variables. CEO tenure has been found to have an impact on corporate strategy making. Thus, we controlled it in terms of the number of years of the CEO’s tenure (Zhang and Rajagopalan, 2010). Previous studies have proved that the concurrent employment of CEOs in two occupations has a significant correlation with corporate strategy. And, in the research of strategic leadership, concurrent serving as CEO is widely used as control (Zhang and Rajagopalan, 2010; Gamache et al., 2020). Therefore, we coded the year when the CEO serves as the chairman of the board at the same time as 1 and 0 in other cases. For the measurement of the size of the board of directors, this article mainly draws on the method of Zhang, and the number of directors of the board of directors in that year represents the size of the board of directors in that year (Zhang, 2006). Previous studies have found that the independence of the board of directors will significantly affect the effectiveness of corporate governance and corporate performance. Therefore, this article takes the independence of the board of directors (Idr) as a control variable. We represent the independence of the board of directors as the percentage of independent directors of the company to the total number of independent board of directors. The firm size represents the uncertainty of enterprise management and the ability to support new product development. We controlled it by the logarithm of total assets. We measured leverage ratio (Lev) by using the ratio of total liabilities to total assets. R&D investment will have a significant impact on corporate strategy; thus, we controlled it in terms of the ratio of enterprise R&D expenditure to sales revenue. Roe is an important feature of an enterprise and represents the financial risk of an enterprise. We measured it by the proportion of corporate assets and corporate liabilities. The higher the concentration of equity, the more the interests of major shareholders will be affected by the company’s future development. This paper selects the share of the largest shareholder as a measure of the company’s shareholding concentration (Herf) (Ulrike and Devin, 2007; Halvorson and Higgins, 2013; Johnson et al., 2013).

### Model Specification

We first conducted a Hausman test, which showed the fixed-effect model was suitable for our model. Regression equation is shown as follows:

Regression equation (1) was established to test the relationship of the control variables with corporate strategic change. And regression (2) was established to test the relationship of two CEO regulatory focuses and the level of corporate strategic change:


(1) $$Sc=\beta_{0}+\beta_{1}Pro+\beta_{2}Pre+\beta_{3}Controls+\sum Industry+\sum year+\varepsilon$$

(2) $$Sc=\beta_{0}+\beta_{1}Pro+\beta_{2}Ferror+\beta_{3}Ferror\#Pro+\beta_{4}Controls+\sum Industry+\sum year+\varepsilon$$

Regression equation (3) was established to test the moderating effect in the relationship of two CEO regulatory focuses and the level of corporate strategic change:


(3) $$Sc=\beta_{0}+\beta_{1}Pre+\beta_{2}Ferror+\beta_{3}Ferror\#Pre+\beta_{4}Controls+\sum Industry+\sum year+\varepsilon$$

The Sc is the level of strategic change. The Pro and Pre are the levels of CEO promotion focus and CEO prevention focus. Ferror is the analysts’ optimism bias. Controls are the control variables. Industry and year represent the dummy variable of industry and year, respectively. β_1_, β_2_, β_3,_ and β_4_ represent the coefficient of each variable [56].

## Results

### Descriptive Analysis

Descriptive statistics and inter-correlations are shown in Table 1. As prior studies, promotion and prevention focuses are independent constructs; in our sample, they are correlated at r = −.01, which is consistent with prior work on CEO regulatory focus [16]. Statistics for year dummy and industry dummy are not shown. The results of Pearson correlation coefficient show that the correlation coefficients between the variables were below.6. On this basis, it can be preliminarily judged that there were no multiple collinearities among the variables [57, 58].

**TABLE 1:** Descriptive statistics and correlations.

Variable	Mean	SD	1	2	3	4	5	6	7	8	9	10	11	12	13
Pro	1.79	0.59													
Pre	0.40	0.29	–0.01												
Sc	0.57	0.26	0.02	–0.03											
Ferror	2.76	5.53	–0.04	–0.04	0.06										
Tenure	4.09	3.06	0.01	0.02	0.01	–0.03									
Dual	0.25	0.44	–0.06	–0.16	0.09	0.05	0.14								
Board	8.71	1.55	0.08	0.09	–0.07	–0.08	0.03	–0.17							
Idr	0.37	0.05	–0.02	–0.04	0.09	0.09	–0.04	0.10	–0.44						
Size	22.03	1.12	0.04	0.21	–0.06	–0.07	0.04	–0.24	0.30	–0.05					
Lev	0.39	0.20	0.08	0.21	–0.12	0.04	–0.06	–0.22	0.25	–0.05	0.54				
Rd	0.04	0.03	–0.14	–0.22	0.26	0.07	0.15	0.21	–0.21	0.12	–0.27	–0.37			
Roe	0.08	0.08	0.03	0.09	–0.09	–0.34	0.05	–0.09	0.03	–0.05	0.21	–0.05	–0.11		
Pay	15.29	0.64	0.06	0.03	0.01	–0.09	0.01	–0.11	0.24	–0.04	0.48	0.17	–0.06	0.25	
Herf	33.80	13.54	0.06	0.05	–0.06	0.04	–0.08	–0.08	0.02	0.03	0.12	0.03	–0.11	0.07	–0.05

*n = 2,284. Correlations greater than 0.04 at p < 0.05, greater than 0.05 at p < 0.01, greater than 0.06 at p < 0.001.*

### Empirical Results

Table 2 presents the analyses of fixed-effect analyses. Hypothesis (H1) predicted CEO promotion focus would be positively associated with corporate strategic change. Model 1 includes only our control variables; Model 2 estimates the relationship between CEO promotion focus and corporate strategic change. The coefficient for CEO promotion focus in Model 2 provides support for Hypothesis (H1) (β = 0.035; *p* = 0.001). This finding suggests that the level of corporate strategic change increases as CEO promotion focus increases. This means that, for every additional unit in the degree of CEO promotion focus, corporate strategic change will increase by about.035 units. Hypothesis (H2) predicted that CEO prevention focus would be negatively associated with the level of corporate strategic change. Model 2 estimates the predicted relationship. The coefficient for CEO prevention focus in Model 2 provides support for Hypothesis (H2) (β = −0.054; *p* = 0.000). This means that, for every additional unit in the degree of CEO promotion focus, corporate strategic change will decrease by about.054 units.

**TABLE 2:** The effect of CEO regulatory focus and analysts’ optimism bias on strategic change.

	Depnedent Variables: Corporate Strategic Change		
Variables	Model (1)	Model (2)	Model (3)
Pro		0.035***	0.030***
		(0.008)	(0.009)
Pre		−0.054***	−0.049***
		(0.021)	(0.022)
Ferror			−0.002**
			(0.003)
Pro#Ferror			0.002***
			(0.001)
Pre#Ferror			−0.004**
			(0.003)
Tenure	0.002	−0.001**	−0.001**
	(0.003)	(0.002)	(0.002)
Dual	0.002	0.008*	0.008**
	(0.003)	(0.015)	(0.015)
Board	0.003***	−0.006***	−0.007***
	(0.001)	(0.005)	(0.005)
Idr	0.018	−0.063**	−0.069***
	(0.025)	(0.139)	(0.139)
Size	−0.001	0.005**	0.003**
	(0.001)	(0.009)	(0.009)
Lev	−0.088***	−0.083*	−0.07*
	(0.008)	(0.044)	(0.044)
Rd	−0.376***	0.023**	0.014**
	(0.036)	(0.204)	(0.203)
Roe	−0.004	−0.144*	−0.137***
	(0.011)	(0.061)	(0.063)
Pay	−0.002	−0.021	−0.02**
	(0.002)	(0.014)	(0.014)
Herf	−0.001	0.001	0.001***
	(0.001)	(0.001)	(0.001)
Constant	0.103*	0.756**	0.776**
	(−0.054)	(0.299)	(0.299)
Year dummy	Control	control	Control
Industry Dummy	Control	control	Control
N	2554	2554	2554
R-Squared	0.107	0.152	0.179

(1) *p < 0.05, **p < 0.01, and ***p < 0.001. (2) Standard errors are in parentheses.

**FIGURE 1:**
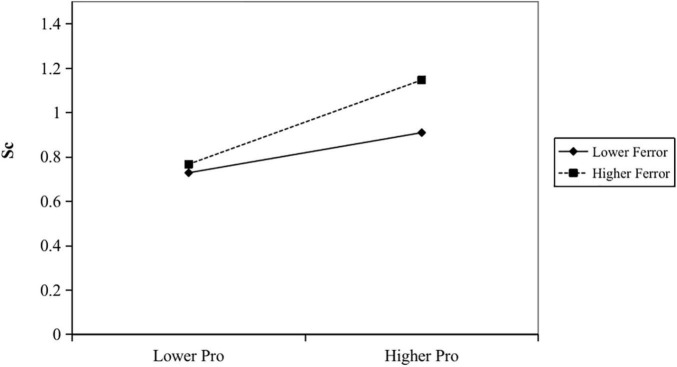
CEO promotion focus and strategic change: the moderating effect of analysts’ optimism bias.

**FIGURE 2:**
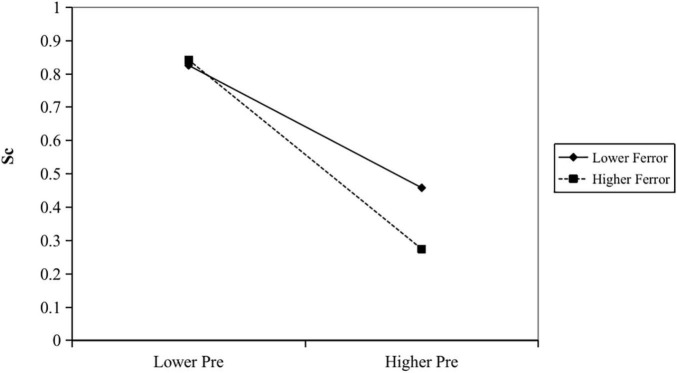
CEO prevention focus and strategic change: the moderating effect of analysts’ optimism bias.

Hypothesis (H3) predicted that analysts’ optimism bias in earning forecasts amplifies the positive relationship between promotion focus and the level of corporate strategic change. In Model 3 of Table 3, the interaction of CEO promotion focus and analysts optimism bias is positive and significant (β = 0.002; *p* = 0.000). The result demonstrates that the analysts’ optimism bias positively moderates the relationship between CEO promotion focus and corporate strategic change. Finally, Hypothesis (H4) predicted that that analysts’ optimism bias in earning forecasts would positively moderate the negative relationship between CEO prevention focus and analysts’ optimism bias. Also, in Model 3 of Table 3, the interaction of CEO prevention focus and analysts’ optimism bias is negative and significant (β = −0.004; *p* = 0.012). The result demonstrates that the analysts’ optimism bias positively moderates the relationship between CEO prevention focus and corporate strategic change. To further explore the moderating effect, we plotted the Figure 1 and Figure 2.

**TABLE 3:** The effect of CEO regulatory focus indicator and analysts’ optimism bias on strategic change.

Depnedent Variables: Corporate Strategic Change	
CEO Regulartory Focus	0.037***
	(0.009)
Ferror	−0.001
	(0.001)
CEO Regualatory Focus#Ferror	0.003**
	(0.001)
Tenure	−0.001
	(0.002)
Dual	0.014
	(0.016)
Board	−0.018***
	(0.006)
Idr	−0.047
	(0.155)
Size	0.013
	(0.010)
Lev	−0.0853*
	(0.050)
Rd	0.0714
	(0.220)
Roe	−0.220***
	(0.073)
Pay	0.020
	(0.016)
Herf	−0.001
	(0.001)
Year dummy	control
Industry Dummy	control
N	2286
R-Squared	0.042

(1) *p < 0.05, **p < 0.01, and ***p < 0.001. (2) Standard errors are in parentheses.

### Robust Test

#### Propensity Score Matching Method

To alleviate the potential endogeneity that CEOs might be selected or select to demonstrate the characteristics that are consistent with the company, we drew from the previous studies, using the PSM method as an alternative measurement for our explanatory variable. Also, our result suggests, under the condition of higher analysts’ bias, CEO with higher promotion focus would more likely to make firm strategic change decision, and CEO with higher prevention focus would be more unwilling to make firm strategic change decision. To validate such relative differences between CEO with higher promotion focus and CEO with higher prevention, we draw from the practice of Mount and Baer, using the treatment indicator of CEO regulatory focus rather than measuring two regulatory focuses, respectively (Mount and Baer, 2021) and reran the whole model. To generate the sample of CEO regulatory focus, we used the propensity score matching (PSM) method. Firstly, we defined CEO promotion focus as the treatment group and CEO prevention focus as the control group. And then, if the CEO had a higher promotion focus than prevention focus, we defined our promotion-focused CEOs treatment group as 1; otherwise, the prevention-focused CEOs control group was 0. Furthermore, we matched observations between the treatment and the control group using a logistic regression through the effects psmatch command in STATA 15.1 by regressing the control variables specified above on the treatment variable. The result shows that the treatment group and the control group are not significantly different, indicating the success of this approach.

Next, we used the treatment indicator that represents the CEO regulatory focus as our sample data and reran the whole model. The results are provided in Table 3; the coefficient for the CEO regulatory focus is significant and positive (β = 0.037; *p* = 0.000), which indicates that CEO promotion focus is positively influenced by the firm strategic change relative to CEO prevention focus. And the coefficient for the interaction between CEO regulatory focus and analysts’ optimism bias is significant and positive (β = 0.003; *p* = 0.019), which indicates that promotion focus relative to prevention focus is positively moderated by analysts’ optimism bias. The results support our hypothesis (Ke and Yu, 2006; Kark and Van Dijk, 2007).

#### Alternative Measures for Corporate Strategic Change

In order to further solve the endogenous problem of possible reverse causality between CEO regulatory focus and strategic change, we used the difference between the average value of the six indicators adjusted by the industry median in the 5th year (t + 3) and the 1st year (t − 2) of CEO tenure to measure the corporate strategic change. Then, we reran the whole model, and the results show in the Table 4 which are consistent with our primary analysis.

**TABLE 4:** Aternative measures for corporate strategic change.

Dependent Variables: Corporate Strategic Change			
Variables	Model (1)	Model (2)	Model (3)
Pro		0.040***	0.036***
		(0.00746)	(0.008)
Pre		−0.061***	−0.074***
		(−0.024)	(0.022)
Ferror			−0.005**
			(0.002)
Pro#Ferror			0.003**
			(0.001)
Pre#Ferror			−0.005*
			(0.003)
Tenure	−0.001	−0.001	0.001
	(0.002)	(0.002)	(−0.002)
Dual	0.008	0.008	0.0248
	(0.0150)	(0.014)	(−0.016)
Board	−0.006***	−0.006***	0.003***
	(0.005)	(0.005)	(−0.006)
Idr	−0.090	−0.068	0.244
	(0.140)	(0.139)	(−0.155)
Size	0.005	0.005	−0.001
	(0.009)	(0.009)	(−0.001)
Lev	−0.094***	−0.084*	−0.002***
	(0.045)	(0.044)	(−0.050)
Rd	−0.058***	0.0342	−0.288***
	(0.205)	(0.204)	(0.232)
Roe	−0.127	−0.138***	−0.002
	(0.062)	(0.070)	(0.072)
Pay	−0.018	−0.020	−0.002
	(0.014)	(0.013)	(−0.016)
Herf	0.0015	0.001	0.001*
	(0.001)	(0.001)	(−0.001)
Constant	0.739**	0.707**	0.507***
	(−0.3)	(−0.298)	(−0.3)
Year dummy	control	control	Control
Industry Dummy	control	control	control
N	2554	2554	2554
R-Squared	0.144	0.148	0.124

(1) *p < 0.05, **p < 0.01, and ***p < 0.001. (2) Standard errors are in parentheses.

## Discussion

### Theoretical Contributions

Our work makes several contributions. First, this study contributes to the upper echelons theory by incorporating the CEO regulatory focus into firm strategic change. The psychological processes by which CEOs influence corporate strategy remain largely a “black box.” Upper echelons theory attempts to delve into this “black box,” which incorporates a retrospective look at CEO behavior. Hambrick (2007) notes that understanding the psychological mechanisms that drive executive behavior is important for future upper echelons theory research. The incorporation of regulatory focus theory into upper echelons theory research is consistent with previous upper echelons theory researchers’ calls for the introduction of tools and concepts used by psychologists. By demonstrating how the CEO promotion and prevention foci differentially influence the CEO’s goal-seeking preferences and strategic change in the firm, this study provides a further exploration of the “black box” of CEO decision making. It also responds to Chiaburu et al.’s (2010) call for more empirical research to further develop upper echelons theory.

Second, we introduce the regulatory focus theory into the research topic of strategic change, which enriches the research on the antecedents of strategic change. Strategic change involves immediate and discontinuous shifts in strategy, power, structure, and control throughout an organization (Virany et al., 1992), and has been an important issue in organizational and strategy research. Previous scholars have mainly examined the antecedents of organizational strategic change from two aspects: the internal organization (resources, cognition, and competitiveness, etc.) and the organizational environment (intensity of competition in the industry, regulatory changes, technological changes, etc.) (Zajac et al., 2000). Specifically, from the perspective of cognition, some scholars explore how factors such as the age, tenure, and educational background of the senior management team affect the way they perceive and interpret the external environment, and ultimately affect the process mechanism of strategic change (Boeker, 1997; Barker et al., 2001). Another group of scholars studies the influence of CEO origin, tenure, personality, and succession style on strategic change from the perspective of the CEO (Pitcher et al., 2000). However, little research has been done on the role of CEO regulatory focus in the process of corporate strategic change. This study uses CEO regulatory focus as an antecedent variable, and the research proves that some CEOs choose to adopt a conservative attitude toward strategic change out of their own prevention focus, while others carry out strategic change out of their promotion focus, which enriches the research framework of corporate strategic change.

Third, we contribute to the contextualized strategic leadership research. Nowadays, external governance bodies are playing an increasingly important role in corporate governance. Numerous studies have verified the important role of external governance entities in strategic decision-making (Goranova et al., 2010, 2017; Shi et al., 2020). For example, executives who are very concerned about the future tend to make long-term-oriented investment decisions (Desjardine and Bansal, 2019). On the contrary, in the face of short-term performance pressure from aggressive hedge funds, executives’ propensity to focus on future long-term investments may be dampened. Therefore, a comprehensive understanding of strategic leadership requires us to consider the various actors involved in governance. However, scholars have not yet considered how external governance bodies moderate the impact of executive characteristics on firm choice and behavior. Also, an analyst as an important stakeholder that will indirectly affect corporate strategy making has not been seriously considered on the strategic leadership research. Hence, we chose analysts’ optimism bias in the earning forecasts as our moderating variable to fully understand the analysts’ contextual effect on the relationship between CEO regulatory focus and the level of corporate strategic change. We found that analysts’ optimism bias in earning forecasts would make CEOs with stronger promotion focus more inclined to make corporate strategic change and make CEOs with stronger prevention focus less inclined to make strategic change decision. Our findings further expand the research framework for contextualized strategic leadership.

### Practical Implications

First, this study provides important implications for the CEOs. Our study suggests that managers should be aware of their own nature and avoid motivations arising from their own personality reasons to influence a firm’s strategic decisions. Although strategic change can have positive outcomes, it is also important to guard against CEOs making strategic decisions that cater to their own regulatory focus rather than to the fact. This study demonstrates that CEOs with strong promotion focus will make decisions to implement strategic change to satisfy their own needs for profit and desire for success, even though the decision may not be appropriate for the current state of the firm. Conversely, CEOs with strong prevention focus will take a conservative approach to strategic change, focusing on the current interests of a firm, its shareholders, and other stakeholders, even though strategic change will benefit the firm in the future.

Second, our study has an important implication for the board of directors and a firm’s stakeholders when they are selecting a CEO. For example, the board of directors can choose a CEO based on the current situation of the company, and a CEO with a strong promotion focus is more appropriate when the company is facing fierce competition and needs to actively seek changes to capture more market share and gain additional profits. When the board of directors believes that the company should grow steadily and meet the current interests of employees and shareholders, a CEO with a strong prevention focus is more effective.

Third, this study provides a reference for investors. CEOs will make different strategic decisions in the face of varying degrees of optimistic bias in analysts’ earnings forecasts. Therefore, investors should choose carefully when making secondary market investments. Investment choices should not be made solely on the basis of analysts’ earnings forecasts but should fully understand the CEO’s personal psychological traits in order to make accurate judgments about the future development of the company.

### Limitations and Future Research

This study has several limitations. First, in terms of research methods, the measurement of CEO regulatory focus in this article is mainly based on text analysis. Future research can adopt a variety of methods to measure CEO regulatory focus, such as questionnaire measurement and case analysis. In the sample selection, this article does not distinguish between specific industries. Future research can subdivide industries to study the relationship between CEO regulatory focus and strategic change. And the measure of regulatory focus in this paper might capture a level beyond the CEO. A future study could use a more accurate sample to measure, like CEOs’ personal interview data.

Furthermore, this article only considers the impact of the CEO’s regulatory focus on a single chain of corporate strategic changes. Future research can add the variable of corporate sustainability performance to explore the mechanism of the impact of CEO regulatory focus on corporate performance. At the same time, institutional logic has always been an important part of strategic change research. Future research can be integrated with institutional theory, for example, to explore how the CEO’s regulatory focus is affected by the external institutional environment.

## Data Availability Statement

The original contributions presented in the study are included in the article/supplementary material, further inquiries can be directed to the corresponding author/s.

## Author Contributions

CH and WZ: conceptualization, validation, and methodology. WZ: software, formal analysis, investigation, resources, data curation, writing—original draft preparation, and visualization. CH: writing—review and editing and supervision. Both authors have read and agreed to the published version of the manuscript.

## Conflict of Interest

The authors declare that the research was conducted in the absence of any commercial or financial relationships that could be construed as a potential conflict of interest.

## Publisher’s Note

All claims expressed in this article are solely those of the authors and do not necessarily represent those of their affiliated organizations, or those of the publisher, the editors and the reviewers. Any product that may be evaluated in this article, or claim that may be made by its manufacturer, is not guaranteed or endorsed by the publisher.

## References

[B1] Abernethy M. A. Dekker H. C. Grafton J. (2020). The influence of performance measurement on the processual dynamics of strategic change. *Manag. Sci.* 67 640–659.

[B2] Adner R. Helfat C. E. (2003). Corporate effects and dynamic managerial capabilities. *Strateg. Manag. J.* 24 1011–1025.

[B3] Ajinkya B. Bhojraj S. Sengupta P. (2005). The association between outside directors, institu-tional investors and the properties of management earnings forecasts. *J. Account. Res.* 43 343–376.

[B4] Alexander K. Thomas P. (2014). Analyst herding and investor protection: a cross-country study. *Appl. Financ. Econ.* 24 533–542.

[B5] Bali T. G. Brown S. J. Tang Y. (2017). Is economic uncertainty priced in the cross-section of stock returns? *J. Financ. Econ.* 126 471–489. 10.1016/j.jfineco.2017.09.005

[B6] Barker I. I. I. V. L. Patterson P. W. Jr. Mueller G. C. (2001). Organizational causes and strategic consequences of the extent of top management team replacement during turnaround at-tempts. *J. Manag. Stud.* 38 234–269.

[B7] Boeker W. (1997). Strategic change: the influence of managerial characteristics and organizational growth. *Acad. Manag. J.* 40 152–170. 10.5465/257024

[B8] Boyne G. A. Meier K. J. (2009). Environmental change, human resources and organizational turnaround. *J. Manag. Stud.* 46 835–863. 10.1097/00005110-200511000-00004 16282825

[B9] Brockner J. Higgins E. T. Low M. B. (2004). Regulatory focus theory and the entrepreneurial process. *J. Bus. Ventur.* 19 203–220. 10.2147/PRBM.S337464 35027854 PMC8752367

[B10] Chen Q. Jiang W. (2006). Analysts’ weighting of private and public information. *Rev. Financ. Stud.* 19 319–355. 10.1093/rfs/hhj007

[B11] Chen X. Cheng Q. Lo K. (2010). On the relationship between analyst reports and corporate disclosures: exploring the roles of information discovery and interpretation. *J. Account. Econ.* 49 206–226. 10.1016/j.jacceco.2009.12.004

[B12] Chiaburu D. S. Sawyer K. B. Thoroughgood C. N. (2010). Transferring more than learned in training: employees’ and managers’ (over)generalization of skills. *Int. J. Sel. Assess.* 18 380–393. 10.1111/j.1468-2389.2010.00520.x

[B13] Cho H. Kim R. Johnson D. T. (2019). Analysts’ optimism and stock crash risk. *Manage. Financ.* 46 323–343. 10.1108/mf-11-2018-0540

[B14] Crowe E. Higgins E. T. (1997). Regulatory focus and strategic inclinations: promotion and prevention in decision-making. *Organ. Behav. Hum. Decis. Process.* 69 117–132. 10.1177/0146167214566188 25575871

[B15] Cuculiza C. Antoniou C. Kumar A. (2020). Terrorist attacks, analyst sentiment, and earnings forecasts. *Manag. Sci.* 67 2579–2608.

[B16] Daily C. M. Dan R. D. (2003). Conflicts of interest: a corporate governance pitfall. *J. Bus. Strategy* 24 631–636.

[B17] Desjardine M. Bansal P. (2019). One step forward, two steps back: how negative external evaluations can shorten organizational time horizons. *Organ. Sci*. 30. 10.1287/orsc.2018.1259 19642375

[B18] Dowen R. J. (1989). What are analysts’ forecasts worth? one-period growth expectations and subsequent stock returns. *Financ. Anal. J.* 45 1134–1152.

[B19] Eggers J. P. Kaplan S. (2009). Cognition and renewal: comparing CEO and organizational ef-fects on incumbent adaptation to technical change. *Organ. Sci.* 20 461–477. 10.1287/orsc.1080.0401 19642375

[B20] Finkelstein S. Hambrick D. C. (1990). Top-management-team tenure and organizational out-comes: the moderating role of managerial discretion. *Adm. Sci. Q.* 35 484–503.

[B21] Förster J. Higgins E. T. (2005). How global versus local perception fits regulatory focus. *Psychol. Sci.* 16 631–636. 10.1111/j.1467-9280.2005.01586.x 16102066

[B22] Förster J. Higgins E. T. Bianco A. T. (2003). Speed/accuracy decision in task performance: built-in-trade-off or separate strategic concerns. *Organ. Behav. Hum. Decis. Process.* 90 148–164. 10.1016/s0749-5978(02)00509-5

[B23] Friedman R. S. Förster J. (2005). Effects of motivational cues on perceptual asymmetry: im-plications for creativity and analytical problem solving. *J. Pers. Soc. Psychol.* 88 263–275. 10.1037/0022-3514.88.2.263 15841858

[B24] Gamache D. L. McNamara G. Mannor M. J. (2015). Motivated to acquire? The impact of CEO regulatory focus on firm acquisitions. *Acad. Manag. J.* 58 1261–1282. 10.5465/amj.2013.0377

[B25] Gamache D. L. Neville F. Bundy J. (2020). Serving differently: CEO regulatory focus and firm stakeholder strategy. *Strateg. Manag. J.* 41 1305–1335.

[B26] Gao P. Y. (2008). Keynesian beauty contest, accounting disclosure, and market efficiency. *J. Account. Res.* 46 785–807. 10.1111/j.1475-679x.2008.00295.x

[B27] Gedajlovic E. Lubatkin M. H. Schulze W. S. (2004). Crossing the threshold from founder management to professional management: a governance perspective. *J. Manag. Stud.* 41 899–912.

[B28] Gioia D. A. Chittipeddi K. (1991). Sensemaking and sensegiving in strategic change initiation. *Strateg. Manag. J.* 12 433–448. 10.1002/smj.4250120604

[B29] Goranova M. L. Priem R. L. Ndofor H. A. Trahms C. A. (2017). Is there a “dark side” to monitoring? Board and shareholder monitoring effects on M&A performance ex-tremeness. *Strateg. Manag. J.* 38 2285–2297. 10.1002/smj.2648

[B30] Goranova M. Dharwadkar R. Brandes P. (2010). Owners on both sides of the deal: mergers and acquisitions and overlapping institutional ownership. *Strateg. Manag. J.* 31 1114–1135. 10.1002/smj.849

[B31] Halvorson H. G. Higgins E. T. (2013). Do you play to win—or to not lose? *Harv. Bus. Rev.* 91 117–120. 23451530

[B32] Hambrick D. C. (2007). Upper echelons theory: an update. *Acad. Manag. Rev.* 32 334–343.

[B33] Higgins E. T. (1997). Beyond pleasure and pain. *Am. Psychol.* 52 1280–1300. 9414606 10.1037//0003-066x.52.12.1280

[B34] Hitt M. A. JeanLuc A. Michael H. R. (2021). Strategic management theory in a post-pandemic and non-ergodic world. *J. Manag. Stud.* 58 259–264.

[B35] Hong H. Lim T. Stein J. C. (2000). Bad news travels slowly: size, analyst coverage, and the profitability of momentum strategies. *J. Financ.* 55 265–295.

[B36] Johnson P. D. Smith M. B. Wallace J. C. Hill A. D. Baron R. A. (2015). A review of multilevel regulatory focus in organizations. Journal of Management, 41, 1501–1529. 10.1177/0149206315575552

[B37] Johnson R. E. Steinman L. (2009). Use of implicit measures for organizational research: an empirical example. *Can. J. Behav. Sci.* 41 202–212.

[B38] Johnson R. E. Chang C. H. Meyer T. (2013). Approaching success or avoiding failure? Ap-proach and avoidance motives in the work domain. *Eur. J. Pers.* 27 424–441. 10.1002/per.1883

[B39] Johnson R. E. King D. D. Lin S. H. (2017). Regulatory focus trickle-down: how leader regula-tory focus behavior shapes follower regulatory focus. *Organ. Behav. Hum. Decis. Process.* 140 29–45. 10.1016/j.obhdp.2017.03.002

[B40] Johnson R. E. Lanaj K. Tan J. A. (2012a). *Putting our Trust in Fairness: Justice and Regulatory Focus as Triggers of Trust and Cooperation. Research in Management.* Hartford, CT: In-formation Age Publishing.

[B41] Johnson R. E. Venus M. Lanaj K. (2012b). Leader identity as an antecedent of the frequency and consistency of transformational, consideration, and abusive leadership behaviors. *J. Appl. Psychol.* 97 1262–1272. 10.1037/a0029043 22730903

[B42] Kammerlander N. Burger D. Fust A. (2015). Exploration and exploitation in established small and medium-sized enterprises: the effect of CEOs’ regulatory focus. *J. Bus. Ventur.* 30 582–602. 10.1016/j.jbusvent.2014.09.004

[B43] Kark R. Van Dijk D. (2007). Motivation to lead, motivation to follow: the role of the self-regulatory focus in leadership processes. *Acad. Manag. Rev.* 32 500–528. 10.5465/amr.2007.24351846

[B44] Kark R. Van Dijk D. (2019). Keep your head in the clouds and your feet on the ground: a multi-focal review of leadership-followership self-regulatory focus. *Acad. Manag. Ann.* 13 509–546. 10.5465/annals.2017.0134

[B45] Ke B. Yu Y. (2006). The effect of issuing biased earnings forecasts on analysts’ access to management and survival. *J. Account. Res.* 44 965–999. 10.1111/j.1475-679x.2006.00221.x

[B46] Lanaj K. Chang C. H. Johnson R. E. (2012). Regulatory focus and work-related outcomes: a review and meta-analysis. *Psychol. Bull.* 38 998–1034. 10.1037/a0027723 22468880

[B47] Mintzberg H. (1989). Mintzberg on management: inside our strange world of organizations. *Mintzberg Manag. Inside Strange World Organ.* 16 119–126.

[B48] Mount M. P. Baer M. (2021). CEOs’ regulatory focus and risk-taking when firms perform be-low and above the bar. *J. Manag.*

[B49] Nadkarni S. Herrmann P. (2010). CEO personality, strategic flexibility, and firm performance: the case of the indian business process outsourcing industry. *Acad. Manag. J.* 53 1050–1073. 10.5465/amj.2010.54533196

[B50] North D. C. (1999). Dealing with a non-ergodic world: institutional economics, property rights and the global environment. *Duke Environ. Law Policy Forum* 10 1–12.

[B51] Pitcher P. Chreim S. Kisfalvi V. (2000). CEO succession research: methodological bridges over troubled waters. *Strateg. Manag. J.* 21 625–648. 10.1002/(sici)1097-0266(200006)21:6<625::aid-smj107>3.0.co;2-a

[B52] Rajagopalan N. Spreitzer G. M. (1997). Toward a theory of strategic change: a multi-lens per-spective and integrative framework. *Acad. Manag. Rev.* 22 51–55. 10.5465/ambpp.1996.4978183

[B53] Shi W. Connelly B. L. Hoskisson R. Ketchen D. (2020). Portfolio spillover of institu-tional inves- tor activism: an awareness-motivation-capability perspective. *Acad. Manag. J.* 63 1865–1892. 10.5465/amj.2018.0074

[B54] Sofus P. T. M. (2008). More than words: quantifying language to measure firms’ fundamentals. *J. Financ.* 63 437–1467.

[B55] Sonnenfeld J. Kusin M. Walton E. (2013). What CEOs really think of their boards. *Harv. Bus. Rev.* 91 98–106.23898736

[B56] Tumasjan A. Braun R. (2012). In the eye of the beholder: how regulatory focus and self-efficacy interact in influencing opportunity recognition. *J. Bus. Ventur.* 27 622–636. 10.1016/j.jbusvent.2011.08.001

[B57] Tuncdogan A. Boon A. D. Mom T. (2016). Management teams’ regulatory foci and organiza-tional units’ exploratory innovation: the mediating role of coordination mechanisms. *Long Range Plann.* 50 621–635. 10.1016/j.lrp.2016.11.002

[B58] Ulrike M. Devin S. (2007). Are investors naive about incentives? *J. Financ. Econ.* 85 457–489. 10.1037/1076-898X.12.3.155 16953742

[B59] Virany B. Tushman M. L. Romanelli E. (1992). Executive sucession and organization outcomes in turbulent environments: an organization learning approach. *Organ. Sci.* 3 72–91. 10.1287/orsc.3.1.72 19642375

[B60] Wallace J. C. Little L. M. Hill A. D. (2010). CEO regulatory foci, environmental dynamism, and small firm performance. *J. Small Bus. Manag.* 48 580–604. 10.1111/j.1540-627x.2010.00309.x

[B61] Wowak A. J. Hambrick D. C. (2010). A model of person-pay interaction: how executives vary in their responses to compensation arrangements. *Strateg. Manag. J.* 31 803–821.

[B62] Wu C. McMullen J. S. Neubert M. J. (2007). The influence of leader regulatory focus on em-ployee creativity. *J. Bus. Ventur.* 23 587–602. 10.1201/9781315152189-2 29787190

[B63] Zajac E. J. Kraatz M. S. (1993). A diametric forces model of strategic change: assessing the an-tecedents and consequences of restructuring in the higher education industry. *Strateg. Manag. J.* 14 83–102. 10.1002/smj.4250140908

[B64] Zajac E. J. Kraatz M. S. Bresser R. (2000). Modeling the dynamics of strategic fit: a normative approach to strategic change. *Strateg. Manag. J*. 21, 429–453.

[B65] Zhang Y. (2006). The presence of a separate COO/president and its impact on strategic change and CEO dismissal. *Strateg. Manag. J.* 27 283–300. 10.1002/smj.517

[B66] Zhang Y. Rajagopalan N. (2010). Once an outsider, always an outsider? CEO origin, strategic change, and firm performance. *Strateg. Manag. J.* 1 334–346. 10.1002/smj.812

